# Evaluating Molecular Mechanism of Hypotensive Peptides Interactions with Renin and Angiotensin Converting Enzyme

**DOI:** 10.1371/journal.pone.0091051

**Published:** 2014-03-06

**Authors:** Rong He, Rotimi E. Aluko, Xing-Rong Ju

**Affiliations:** 1 College of Food Science and Engineering, Nanjing University of Finance and Economics, Nanjing, People’s Republic of China; 2 The School of Food Science and Technology, Jiangnan University, Wuxi, People’s Republic of China; 3 Department of Human Nutritional Sciences and the Richardson Centre for Functional Foods and Nutraceuticals, University of Manitoba, Winnipeg, Manitoba, Canada; Max-Delbrück Center for Molecular Medicine (MDC), Germany

## Abstract

Our previous study showed that three rapeseed protein-derived peptides (TF, LY and RALP) inhibited the *in vitro* activities of angiotensin converting enzyme (ACE) and renin. Oral administration of these peptides to spontaneously hypertensive rats led to reductions in systolic blood pressure. In the present work, we examined the potential molecular mechanisms responsible for the ACE- and renin-inhibitory activities of these peptides. Enzyme inhibition kinetics showed competitive, non-competitive and mixed-type peptide-dependent inhibition of renin and ACE activities. Intrinsic fluorescence intensity data showed that LY and RALP have stronger binding effects on ACE molecule compared to that of TF. LY and RALP showed the highest inhibition of ACE and renin activities, respectively. Circular dichroism data showed that the inhibitory mechanism involved extensive peptide-dependent reductions in α-helix and β-sheet fractions of ACE and renin protein conformations. Molecular docking studies confirmed that the higher renin-inhibitory activity of RALP may be due to formation of several hydrogen bonds (H-bonds) with the enzyme’s active site residues. The rapeseed peptides inhibited renin and ACE activities mostly through binding to enzyme active site or non-active sites and forming extensive H-bonds that distorted the normal configuration required for catalysis. Data presented from this work could enhance development of highly potent antihypertensive natural peptides or peptidomimetics.

## Introduction

Renin and angiotensin-I converting enzyme (ACE) are the two key enzymes that regulate the renin-angiotensin system (RAS) and are important determinants of blood pressure and fluid homeostasis [1]. Renin cleaves angiotensinogen to yield angiotensin-I, which is subsequently converted by the action of ACE to angiotensin-II, a potent vasoconstrictor that up-regulates blood pressure. Therefore, simultaneous inhibition of renin and ACE activities would prevent the formation of both angiotensin-I and angiotensin-II, which produces a more efficient regulation of RAS when compared to the use of individual enzyme inhibitors alone [2]. The simultaneous inhibition of renin and ACE activities could provide a new alternative way to treat hypertension efficiently without severe negative side effects [3].

As an aspartyl protease, renin contains two catalytic aspartic acid residues (Asp32 and Asp215) that are located in the active site cleft and can accommodate seven amino acid units of the substrate (angiotensinogen). Renin’s catalytic activity involves cleavage of the peptide bond between Leu10 and Val11 of angiotensinogen to generate angiotensin-I [4], [5]. On the other hand, ACE is a zinc-dependent dipeptidyl carboxypeptidase that is composed of two homologous domains (N and C domain) [6]. The C-domain has been shown to be the dominant angiotensin-I converting site with a conserved HEXXH zinc-binding motif for controlling blood pressure and cardiovascular functions [7]. Therefore, inhibitors can cause losses in enzyme activities by occupying the active site of these enzymes and binding to crucial amino acid residues such that substrate binding is prevented. Deactivation of ACE and renin can also be induced by changes in protein conformation around the active site, which occur from molecular collisions with inhibitors. Thus, it is possible to determine the enzyme inactivation mechanisms by analyzing the structural consequences of enzyme-inhibitor interactions. Knowledge of the mechanism of peptide-induced inhibition of enzyme activity could enhance the design of new but potent blood pressure-reducing drugs that are based on ACE and renin protein conformational changes.

The interest in bioactive peptides as agents for the control of hypertension continues to increase and our previous study has confirmed that rapeseed protein-derived peptides (Thr-Phe, Leu-Tyr and Arg-Ala-Leu-Pro) possess dual *in vitro* inhibitions of renin and ACE activities [8]. We also demonstrated the blood pressure-reducing effects of these peptides after oral administration to spontaneously hypertensive rats [8], which indicates physiological relevance. In the current study, we examined the interactions of these rapeseed protein-derived peptides with renin and ACE using techniques that include enzyme inhibition kinetics, conformational analysis and molecular docking. The work was aimed at elucidating how the rapeseed peptides exert their antihypertensive effects and the potential molecular mechanism involved in peptide-dependent inactivation of renin and ACE activities.

## Materials and Methods

### Materials

The rapeseed protein-derived peptides Thr-Phe (TF), Leu-Tyr (LY) and Arg-Ala-Leu-Pro (RALP) were synthesized (>95% purity) by GenWay Biotech (GenWay Biotech Inc. San Diego, CA). Human recombinant renin protein (10006217; >99% purity) and renin inhibitor screening assay kit (10006270) were purchased from Cayman Chemicals (Ann Arbor, MI). Rabbit lung ACE (A6778, 98% purity) and N-[3-(2-Furyl) acryloyl]-L-phenylalanyl-glycyl-glycine (FAPGG) were purchased from Sigma-Aldrich (St. Louis, MO). Other analytical grade reagents were obtained from Fisher Scientific (Oakville, ON, Canada).

### Enzyme Kinetics

Kinetics of ACE and renin inhibition was determined using a previously described method [9]. For ACE inhibition, the substrate (FAPGG) concentrations were 0.0625, 0.125, 0.25 and 0.5 mM, while renin substrate concentrations were 1.25, 2.5, 5 and 10 µM. Peptide concentrations used during the assays are shown in Tables 1 and 2 for the kinetics of ACE and renin inhibition, respectively. The modes of ACE and renin inhibition were determined from Lineweaver-Burk plots while inhibition parameters (*V_max_* and *K_m_*) were calculated, respectively, as the Y and X-axis intercepts of the primary plot. *K_i_* was calculated as the X-axis intercept of the line obtained from a secondary plot of Lineweaver-Burk line slopes versus peptide concentrations.

**Table 1:** Kinetics constants of angiotensin converting enzyme-catalyzed reaction at different peptide concentrations.

Catalytic parameter	Control	TF (mM)		LY (mM)		RALP (mM)	
		0.1887	0.7510	0.0509	0.1020	0.1756	0.3292
*V_max_* or *V*′*_max_* (ΔA/min)	0.0151	0.0185	0.0097	0.0142	0.0147	0.0149	0.0137
*K_m_* or *K*′*_m_* (mM)	0.3310	1.0088	0.5546	0.7123	1.3464	0.5692	0.8117
*K_i_* (mM)		12.2726		0.0312		0.1041	

*K_m_* or *K*′*_m_* is Michaelis-Menten constant in the absence (control) or presence of a peptide; *V_max_* or *V*′*_max_* is maximum reaction velocities in the absence (control) or presence of a peptide; *K_i_* is the enzyme-inhibitor dissociation constant.

**Table 2:** Kinetics constants of renin-catalyzed reaction at different peptide concentrations.

Catalytic parameter	Control	TF (mM)		LY (mM)		RALP (mM)	
		0.9387	3.7551	0.8493	1.6986	0.5487	1.097
*V_max_* or *V*′*_max_* (*FI*/min)	38.58	25.89	17.20	17.74	17.63	21.32	18.27
*K_m_* or *K*′*_m_* (µM)	4.78	4.78	4.41	2.45	3.11	3.54	5.02
*K_i_* (mM)		6.3086		2.2422		0.2853	

*K_m_* or *K*′*_m_* is Michaelis-Menten constant in the absence (control) or presence of a peptide; *V_max_* or *V*′*_max_* is maximum reaction velocities in the absence (control) or presence of a peptide; *K_i_* is the enzyme-inhibitor dissociation constant.

### Intrinsic Fluorescence Emission

Fluorescence emission spectra were obtained using the method of Li et al. [10]. Briefly, 25 µL of 1 U/mL ACE (prepared in 50 mM Tris–HCl buffer, pH 7.5, containing 300 mM NaCl) or 25 µL of 250 µg/mL renin (prepared in 50 mM Tris–HCl buffer, pH 8.0, containing 100 mM NaCl) was mixed with 50 µL of peptide solution and 25 µL of the appropriate buffer to yield a 100 µL assay solution. Emission spectra were recorded at 25°C using a micro quartz cell (100 µL capacity) on a JASCO FP-6300 spectrofluorimeter (JASCO, Tokyo, Japan). ACE or renin assay solutions were excited at 280 nm and emission recorded from 290 to 450 nm. Emission spectrum of the respective buffer, peptides and enzymes was subtracted from the emission spectrum of each assay solution.

### Circular Dichroism (CD)

Far-UV CD spectra were obtained using a JASCO J-815 spectropolarimeter (JASCO, Tokyo, Japan) under constant nitrogen flush according to the method of Omoni and Aluko [11]. A 0.5 mm pathlength quartz cell was used for the far UV (190–240 nm) measurements and each final spectrum was the average of three consecutive scans with simultaneous subtraction of the respective buffer, peptides and enzymes spectrum. For determining the peptide-enzyme interaction spectra, 150 µL of 1 U/mL ACE (prepared in 50 mM Tris–HCl buffer, pH 7.5, containing 300 mM NaCl) or 150 µL of 250 µg/mL renin (prepared in 50 mM Tris–HCl buffer, pH 8.0, containing 100 mM NaCl) was mixed with 50 µL of a peptide solution. The far-UV CD spectra in the 200–240 nm range were analyzed for secondary structure fractions using the online DICHROWEB procedure [12].

### Molecular Docking

Molecular docking was performed using the Accelrys Discovery Studio software 2.5 (DS 2.5) according the method of Pan et al. [13]. The structures of LY, TF and RALP were generated with DS 2.5 and energy was minimized with the CHARMm program. For ACE docking, we used 1O8A (PDB), a crystal structure of human ACE bound to lisinopril (ACE-inhibitory drug). A binding site with a radius of 15 Å and coordinates x: 41.268, y: 34.559 and z: 45.393 was created by the removal of lisinopril from ACE structure. Automated molecular docking was performed using the partial flexibility CDOCKER tool of the DS 2.5 software in the presence of cofactors (zinc and chloride ions). For renin docking, a crystal structure of human renin (2V0Z) bound to aliskiren (renin-inhibitory drug) was used. Aliskiren and all the water molecules present in the renin structure (except H_2_O-184 and H_2_O-250) were removed and hydrogen atoms added. A binding site with coordinates x: 7.568, y: 46.092, z: 68.842 and a radius of 10 Å was created around the ligand [14]. Evaluation of the molecular docking was performed according to the scores and binding-energy values in order to obtain the best peptide poses. DS 2.5 software was also used to identify the hydrogen bonds as well as the hydrophobic, hydrophilic, electrostatic, and coordination interactions between residues present within the ACE or renin active site.

### Statistical Analysis

All assays were conducted in triplicate and analyzed using one-way analysis of variance (ANOVA). The mean values were compared using Duncan’s multiple range test and significant differences accepted at *p*<0.05.

## Results and Discussion

### Inhibition Modes of Peptides

**Figure 1:**
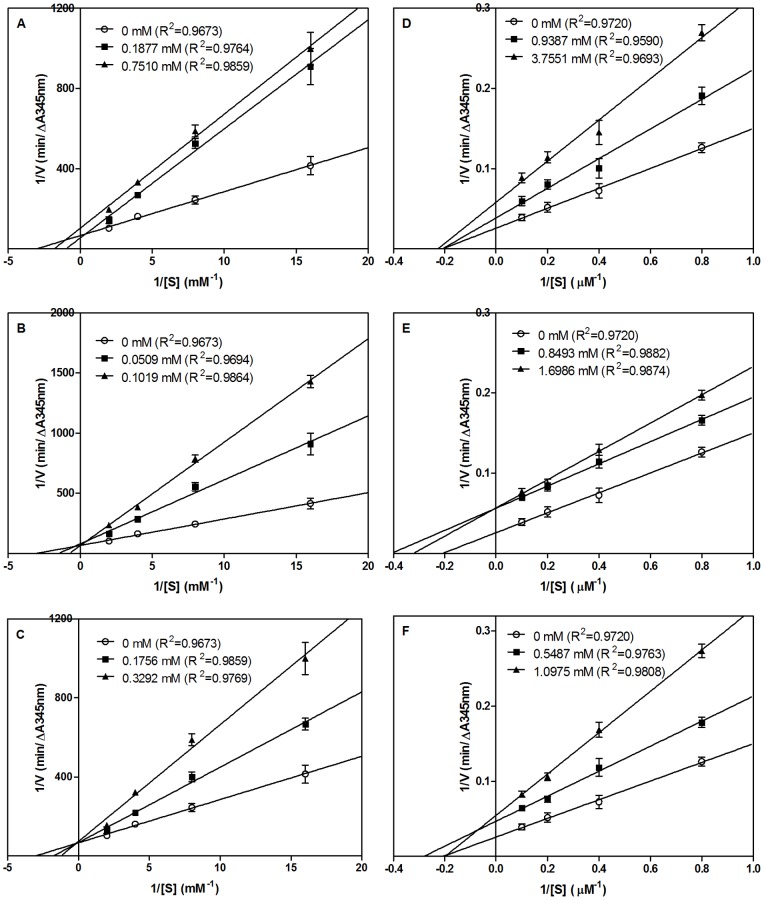
Lineweaver-Burk plots of the inhibition of ACE and renin by peptides. (A) TF, (B) LY, and (C) RALP are at varying concentrations of ACE substrate (0.0625–0.5 mM), and V is rate of reaction (ΔA345 nm/min); (D) TF, (E) LY and (F) RALP are at varying concentrations of renin substrate (1.25–10 µM), and V is rate of reaction (FIU/min).

The IC_50_ values of TF, LY and RALP were previously determined, respectively, to be 0.810, 0.107 and 0.648 mM for ACE inhibition, and 3.061, 1.868 and 0.968 mM for renin inhibition [8]. In this study, Lineweaver-Burk plots were used to evaluate the ACE and renin inhibition modes of the three peptides. As shown in Figure 1A–C, the double reciprocal plots of ACE-catalyzed reactions in the absence and presence of peptides indicate a mostly competitive (LY and RALP) or mixed-type (TF) inhibition of ACE activity. The results suggest that the peptides can bind to ACE only as well as the ACE-FAPGG complex; peptide binding then causes a reduction in ACE catalytic activity. The decreased ACE activity in the presence of peptides was confirmed by the reduced *V_max_* (TF) and increased *K_m_* (TF, LY, and RALP) values as shown in Table 1. The decreased ACE activity in the presence of peptides may be due to active site blockade by TF, LY and RALP that prevented substrate binding or limited substrate access to the active site. TF could also have caused decreased ACE activity through binding to protein sites that are remote from the active site such that the enzyme conformation (and active site configuration) became altered and the substrate could no longer bind efficiently [15]. The results are similar to those reported for peptides such as VEGY [16], and AWLHPGAPKVF [17] that also showed competitive ACE inhibition. Other peptides like TK, RMLGQTP [18], FEDYVPLSCF and FNVPLYE [19] have also been shown to exhibit mixed-type ACE inhibition. The inhibition constant (*K_i_*) is a measure of the peptide binding strength to ACE protein and a lower value usually indicates greater affinity. Therefore, Table 1 shows that LY had the highest binding affinity towards ACE followed by RALP while TF had the lowest affinity; these results are consistent with the IC_50_ values. The *K_i_* values also seem to have a direct relationship with *K_m_* values as shown by the fact that the highest concentration-dependent increase in *K_m_* was associated with LY (88%) in comparison to RALP (42%). Thus LY binding to ACE protein was more effective in reducing catalytic activity when compared to the binding strength of RALP and TF.

The double reciprocal plot showed that TF acted as a non-competitive renin inhibitor (Figure 1D) while LY (Figure 1E) and RALP (Figure 1F) showed uncompetitive and mixed-type inhibitors. Thus, the results suggest that TF has no binding affinity for the active site of renin whereas LY and RALP can bind to the active site as well as other regulatory sites on the renin protein. LY and RALP as uncompetitive inhibitors can also bind to the enzyme-substrate complex to prevent catalysis. The results in Figure 1D–F are supported by data shown in Table 2 where the *K_m_* values for TF are similar to that of the uninhibited reaction, which is typical of non-competitive inhibition. In contrast, the *K_m_* values for renin inhibition by LY and RALP are differed from that of the uninhibited reaction, which suggests a mixed-type mode of enzyme inhibition. The *K_i_* values suggest RALP is a more effective renin inhibitor when compared to LY and TF; the order of *K_i_* value is also positively related to peptide IC_50_ values. It was difficult to do a comparative analysis with previous works because there are almost no related renin inhibition kinetics studies with food protein-derived peptides in the literature. However, a previous work has shown that inhibition of *in vitro* renin activity by hempseed protein hydrolysates was mostly of the mixed-type [9].

### Intrinsic Fluorescence and Quenching Mechanism

**Figure 2:**
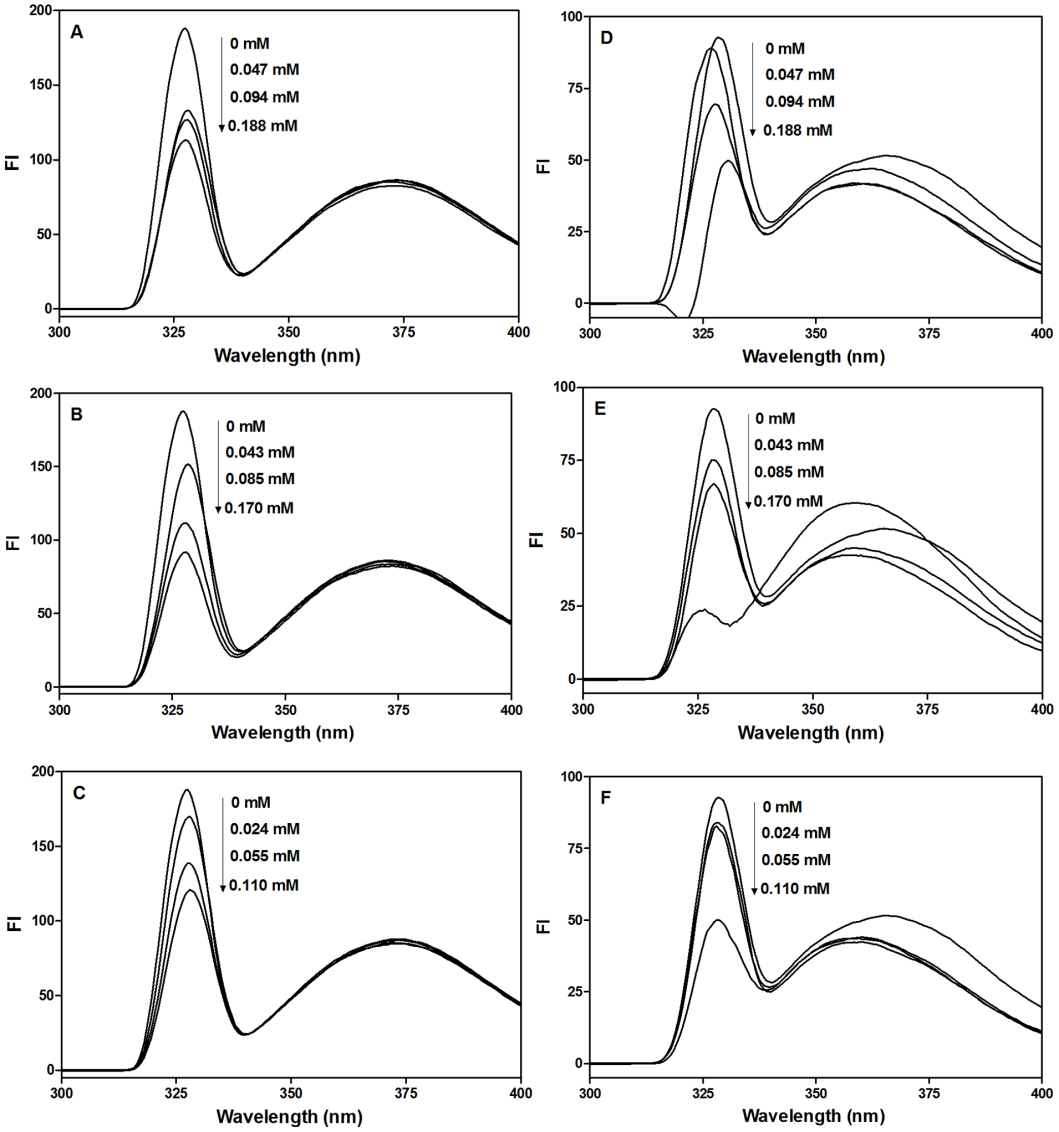
Emission spectra of ACE and renin proteins in the presence of peptides. Results of peptide-ACE interactions are showed in A (TF), B (LY), C (RALP), and that of peptide-renin interactions are D (TF), E (LY), F (RALP). In all cases λ_ex_ = 280 nm.

Fluorescence emission spectroscopy can be used to evaluate the interaction and binding characteristics between peptides and enzyme proteins; information such as binding mechanism, binding constants and the number of binding sites may be obtained. Emission spectra of ACE in the presence of different peptide concentrations are shown in Figure 2A–C. When ACE was excited at 280 nm, a sharp emission maximum was obtained at 327±0.5 nm, which is typical of *Trp* emission but with likely contributions from *Tyr* residues [20]. Addition of peptides resulted in concentration-dependent decreases in FI, which was more pronounced for LY with a 2.05-fold decrease at 0.170 mM when compared to that of ACE alone. This was followed by RALP interaction with ACE while TF produced the least concentration-dependent changes in ACE FI. The changes in ACE FI reflects *K_i_* values as reported in Table 1, which indicates binding affinity increased from TF to LY. The stronger binding effects of LY and RALP may be due to the fact that these peptides contain both bulky and hydrophobic amino acids, which are known to enhance hydrophobic interactions between enzymes and ligands [21]. The results (decreases in FI upon interaction with peptides) indicate partial unfolding of ACE protein structure to produce a more open conformation than the native structure. The open conformation increases exposure of the hydrophobic groups (*Tyr* and *Trp*) in ACE to the hydrophilic environment, which causes FI quenching. In addition to inducing an open protein structure, it is also possible that interaction of peptides with ACE led to shielding of previously exposed (in the native structure) *Tyr* and *Trp* residues, which will reduce the amount of light energy absorbed and hence reduced FI values. Since the wavelength of maximum FI (λ_max_) did not change (∼327 nm), it means that peptide interactions did not change the microenvironment of *Tyr* and *Trp* residues within the ACE protein structure. But the low value of 327 nm suggests that the *Tyr* and *Trp* residues were located in a mostly hydrophobic microenvironment within the ACE protein. The present results agree with a previous report that showed ostrich egg protein-derived peptides caused unfolding of ACE protein, which was accompanied by decreases in ACE FI [22].

Peptide interactions with renin showed maximum FI at 328±0.5 nm when excited at 280 nm; the FI values were gradually decreased as peptide concentration was increased, which indicates modulation of renin protein conformation. However, the largest decrease (74%) in ACE FI was observed when LY was present at 0.170 mM concentration, which suggests greater affinity for renin protein when compared to smaller decreases in FI in the presence of TF (47%) or RALP (46%). As noted above, the decreases in renin FI in the presence of peptides indicate protein conformational changes that exposed hydrophobic groups to the hydrophilic environment. Interactions of TF with renin also led to concentration-dependent shifts in the λ_max_ from 328.5 nm in the absence of peptide to 326, 327 and 330 nm in the presence of 0.047, 0.094 and 0.188 mM peptide concentrations, respectively. Thus at 0.047 and 0.094 mM TF concentrations, there were slight increases in the hydrophobic environment of *Tyr* and *Trp*, which led to the blue shifts in λ_max_. However, at 0.170 mM peptide concentration, there seem to be a change in renin conformation into a more unfolded structure that modified the microenvironment of the *Tyr* and *Trp* residues to have a more hydrophilic character and hence an increase in λ_max_ to 330 nm. For LY, only the 0.170 mM concentration produced a slight blue shift (326 nm) in λ_max_. In contrast to the observed effects of TF and LY on the microenvironment of *Tyr* and *Trp* in renin, RALP did not produce any concentration-dependent change in λ_max_.

### Secondary Structures

**Figure 3:**
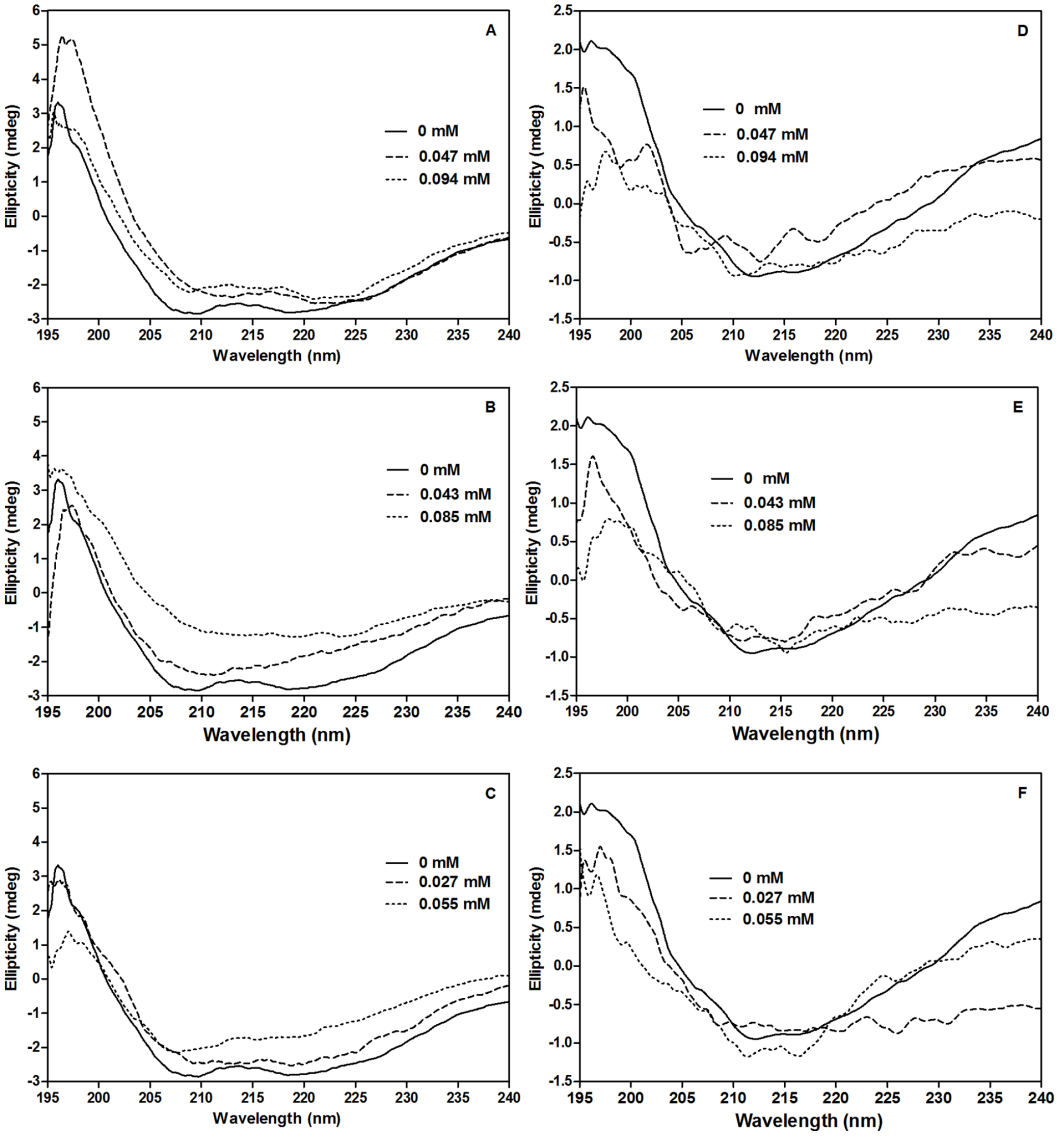
Far-UV circular dichroism spectra of ACE and renin in the presence of peptides. Results of peptide-ACE interactions are showed in A (TF), B (LY), C (RALP), and that of peptide-renin interactions are D (TF), E (LY), F (RALP).

The interactions between ACE or renin and rapeseed peptides were investigated by circular dichroism spectra in the far-UV range of 190–240 nm. As shown in Figure 3A–C, the native ACE protein had a positive band near to 196 nm with a zero crossing at 200 nm and two negative dichroic bands around 208 and 222 nm, indicating a predominantly α-helical structure as it was previously reported for the crystal structure of the human enzyme [23]. After addition of peptides, a red shift of the zero-crossing point occurred and the ellipticity values increased simultaneously, suggesting the loss of α-helical structure with concomitant protein unfolding. The results are consistent with previously reported ACE secondary structure changes induced by egg peptides [24]. In addition, the observed changes in ACE structure were distinctly dependent on the concentration and type of added peptide. For example, at 0.047 mM TF concentration there was mainly an increase in the β-sheet fraction (41.67±2.17%) and decreases in helical and unordered structures (Table 3). However, at 0.094 mM the β-sheet fraction was reduced to 29.17±0.86% (p<0.05), which was accompanied by increased ratio of unordered structure fraction (Table 3). The increased fraction of unordered ACE structure that was induced by TF may be due to constant rate of dynamic ACE-TF collisions. The interaction of LY (0.043 mM) with ACE led mainly to an increase in the fraction of unordered structure (38.03±1.21%) and decreases in α-helix and β-sheet fractions. At 0.085 mM peptide concentration, there were further reductions in α-helix (1.41±0.17%) and β-turn fractions to form a higher content of β-sheet structure (42.80±0.17%). In contrast to LY, the RALP-ACE interactions did not lead to any significant (*p*>0.05) change in unordered structure. However, there were reductions in α-helix fraction while β-sheet and β-turn fractions were significantly (*p*<0.05) increased (Table 3). Overall, the secondary structure changes induced by TF, LY and RALP provide additional potential mechanism through which these peptides inhibit ACE activity.

**Table 3:** The estimated secondary structure composition of enzyme and enzyme-peptide complex.

Treatment	Peptide (µg/mL)	α-helix (%)	β-sheet (%)	β-turn (%)	Unordered (%)
ACE	0	23.55±0.7^A^	24.21±0.28^C^	18.41±0.71^BC^	34.21±0.42^D^
ACE+TF	12.5	16.55±0.49^B^	41.67±2.17^A^	16.97±0.31^C^	22.31±2.25^E^
	25	13.43±0.72^C^	29.17±0.86^B^	17.33±0.64^C^	39.9±1.56^B^
ACE+LY	12.5	13.23±0.96^C^	20.03±0.91^B^	18.07±1.31^C^	38.03±1.21^BC^
	25	1.41±0.17^E^	42.80±0.17^A^	12.17±0.75^D^	44.71±0.69^A^
ACE+RALP	12.5	15.31±0.75^B^	28.97±1.59^B^	19.73±0.31^B^	34.27±1.05^D^
	25	9.97±1.59^D^	31.23±1.59^B^	21.33±0.91^A^	35.67±1.18^CD^
Renin	0	5.63±0.46^B^	46.03±0.71^A^	16.87±0.21^C^	32.17±0.55^E^
Renin+TF	12.5	2.91±0.26^C^	46.97±0.31^A^	21.37±0.06^A^	28.91±0.25^G^
	25	0.95±0.07^D^	7.91±0.85^D^	18.95±0.21^B^	72.25±0.21^C^
Renin+LY	12.5	4.63±0.12^B^	44.41±0.61^B^	19.41±0.17^B^	30.37±0.23^F^
	25	2.63±0.32^C^	4.33±0.32^E^	19.73±1.45^B^	74.87±0.31^B^
Renin+RALP	12.5	10.93±1.15^A^	40.91±1.39^C^	12.23±0.23^E^	34.57±0.06^D^
	25	1.03±0.14^D^	2.21±0.28^F^	13.75±0.07^D^	83.05±0.07^A^

Results are presented as mean ± standard deviation (n = 3) of triplicate determinations. For each enzyme, column values with different letters are significantly different (*p*<0.05).

The native renin protein also displayed a positive ellipticity band near 195 nm but only one negative band from 213 to 217 nm (Figure 3D–F), which indicates a predominantly β-sheet conformation. Therefore, unlike ACE, the protein structural conformation of renin shows 46.03±0.71% β-sheet, 16.87±0.21% β-turn, 5.63±0.46% α-helix and 32.17±0.55% unordered structures (Table 3); the results are similar to those reported for the crystal structure of recombinant human renin [4]. Addition of peptides led to changes in renin protein conformations with TF causing further decreases (*p*<0.05) in α-helix fraction. The most intense change caused by TF was observed at 0.094 mM, which increased the unordered fraction in renin protein structure by a factor of 2.25 while β-sheet fraction was reduced by a factor of ∼6. Similarly, the interactions of LY and RALP (each at respective 0.085 mM and 0.055 mM) with renin also led to significant (*p*<0.05) increases in unordered protein fraction (from 32.17 to 74.87 and 83.05%, respectively) as well as decreases in content of β-sheet and α-helix (Table 3). The results indicate that these peptides inhibited renin activity by increasing formation of a more unordered protein structure that contain a distorted active site, which led to reduced formation of required bonds between catalytic residues and substrate. This may explain why LY and RALP, which caused formation of higher number of unordered protein structure fraction, were more active as renin catalysis inhibitors (lower IC_50_ and *K_i_* values) when compared to TF.

### Molecular Docking

**Figure 4:**
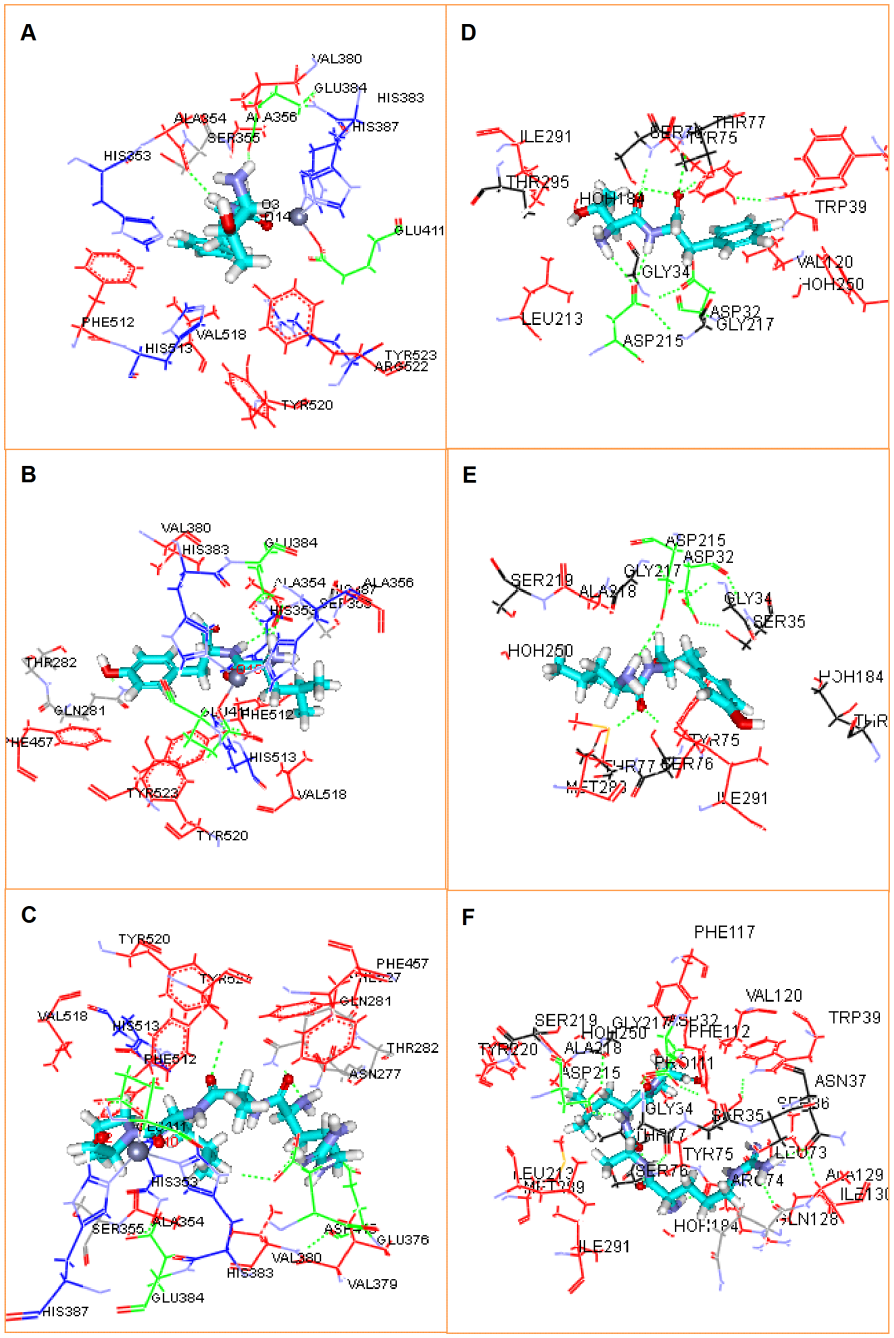
Molecular docking of the peptides at ACE and renin active sites. Results of peptide-ACE interactions are showed in A (TF), B (LY), C (RALP), and that of peptide-renin interactions are D (TF), E (LY), F (RALP). Enzyme hydrophobic residues are represented in red, positively charged residues are represented in blue, negatively charged residues and hydrogen bonds are represented in green, and other residues are represented automatically. (Image obtained with Accelrys DS Visualizer software).

Molecular docking studies of the peptides when present within the ACE catalytic site and in the presence of Zn^2+^ showed the best poses (Figure 4A–C) with potential binding energy values (*E_pot_*) of −31.36, −39.21, and −192.81 kJ/mol for TF, LY and RALP, respectively (Table S1). The best pose for each peptide was stabilised mainly by electrostatic interactions (Table 3 and Table S1), hydrophobic interactions (Table 4) and hydrogen bonds (Table S2) with ACE amino acid residues. Molecular docking of peptides to the active site of ACE demonstrates that three peptides are buried deep inside the hydrophobic pocket, and occupy mainly S1 and S2′ subsites via hydrophobic interactions with Ala354, Glu384, Tyr523 residues for S1 and Gln281, Tyr520, Lys511, His513, His353 residues for S2′ (Figure 4A–C and Table 4). This type of alignment is similar to the observed interactions between lisinopril (ACE-inhibitory drug) and ACE [25]. The presence of multiple H-bond interactions between the peptides and ACE could have contributed to peptide-induced inhibition of enzyme activity by stabilizing the non-catalytic enzyme-peptide complex structure. Our results are similar to those reported in a previous molecular docking work where it was shown that the ACE-inhibitory peptides VPP and IPP formed multiple hydrogen bonds when bound to ACE protein [26]. Although the intrinsic fluorescence data suggested that the interaction of TF with ACE was mainly dynamic without extensive binding between TF and active sites of ACE, the molecular docking data revealed that TF had two H-bond interactions with Ala354 (O, 2.16 Å) and Glu384 (OE2, 2.12 Å) at S1 subsite (Table S2). LY registered an additional H-bond with the Gln281 (HE21, 2.17 Å) at S2′ subsite in comparison to TF (Table S2), which might explain the higher ACE-inhibitory property of LY. RALP also had a higher ACE inhibition when compared to TF, probably as a result of the four H bonds formed with the Gln281 (HE21), Tyr520 (HH) and Asp415 (OD2) at S1 subsite when compared to two for TF (Table S2). The presence of higher number of H-bonds (9 in total) will also explain the greater ACE-inhibitory potency of the commercial antihypertensive drug lisinopril, when compared to the peptides that formed only 2–4 H-bonds (Table S2).

**Table 4:** ACE (PDB: 1O86) and renin (PDB: 2V0Z) residues having at least one atom at a distance of 3.5 Å around the docked peptide.

NO.	ACE residues	TF	LY	RALP	Lisinopril	Renin residues	TF	LY	RALP	Aliskiren
1	Glu162				√	Thr12				√
2	Asn277			√		Gln13				√
3	Gln281		√	√	√	Tyr14				√
4	Thr282		√	√		Val30				√
5	His353	√	√	√	√	Asp32	√	√	√	√
6	Ala354	√	√	√	√	Gly34	√	√	√	√
7	Ser355	√	√	√	√	Ser35		√	√	√
8	Ala356	√	√	√	√	Ser36			√	
9	Val379			√		Asn37			√	
10	Val380	√	√	√	√	Trp39	√		√	√
11	His383	√	√	√	√	Leu73			√	
12	Glu384	√	√	√	√	Arg74			√	√
13	His387	√	√	√	√	Tyr75	√	√	√	√
14	Phe391	√	√			Ser76	√	√	√	√
15	Glu411	√	√	√	√	Thr77	√	√	√	√
16	Asp415		√	√		Pro111			√	√
17	Asp453			√		Phe112	√		√	√
18	Lys454			√		Leu114				√
19	Phe457		√	√	√	Ala115				√
20	Lys511	√			√	Phe117	√		√	√
21	Phe512	√	√	√	√	Val120	√		√	√
22	His513	√	√	√	√	Gln128			√	√
23	Val518	√	√	√	√	Ala129			√	
24	Tyr520	√	√	√	√	ILE130			√	√
25	Arg522	√				Tyr155				√
26	Tyr523	√	√	√	√	Leu213	√		√	√
27	Phe527			√	√	Asp215	√	√	√	√
28						Thr216				√
29						Gly217	√	√	√	√
30						Ala218			√	√
31						Ser219		√	√	√
32						Tyr220			√	
33						Met289			√	
34						Ile291	√	√	√	√
35						Thr295	√	√		√
36						Ala303				√
Total		17	19	23	19		14	11	26	30

The residues around crystallized lisinopril and Aliskiren in the ACE and renin structures are also shown.

In addition to the hydrophobic and H-bond interactions, the interactions between ACE inhibitors and Zn^2+^ at the enzyme active site also play a significant role in modulating catalysis [23]. As shown in Figure 4, all the three peptides were positioned to interact with the active site Zn^2+^ atom, which is in addition to the two zinc-coordinating histidines (His383 and His387) on the helix α13 and Glu411 on the helix α14 of ACE protein [23]. It is believed that the distance between the peptide bond carbonyl oxygen and Zn^2+^ account for the varied degrees of inhibition by peptides, and that the shorter the Zn^2+^ distance to the carbonyl oxygen of the peptide, the greater the degree of inhibition [27]. This might explain the higher ACE inhibition of LY (2.210 Å) and RALP (2.209 Å) with shorter distances when compared to a distance of (2.433 Å) for TF (Table S3). The results obtained in this work are in accord with those reported for Arachin-derived peptides IKP and IEP that showed 2.73 Å and 3.036 Å Zn^2+^ coordination distances with 7 µM and 18 µM IC_50_ values for ACE inhibition, respectively [27].

The docking results for peptide interactions with the active site of renin are shown in Figure 4D–F. The results indicate that RALP showed a better affinity towards renin as evidenced by the lowest binding energy value of −192.88 kJ/mol when compared to −31.46 and −39.91 kJ/mol for TF and LY, respectively (Table S1). In comparison to ACE docking results, renin-bound RALP was also surrounded by several hydrophilic residues such as Asp32, Gly34, Ser76, Thr77, Asp215 and Gly217 (Table 4). Thus, the results indicate that the presence of a positively charged amino acid such as *Arg* may improve formation of multiple interactions and contribute to improved affinity of inhibitory peptides for renin. All the peptides were docked inside the active site of renin protein. For example, RALP occupied seven of the active site pockets (S3′, S2′, S1′, S1, S2, S3, S4) while TF and LY occupied five (S3′, S2′, S1′, S1, S3,) and four pockets (S3′, S2′, S1′, S1, S4), respectively; these active sites have been previously described [4]. The ability of RALP to form higher number of multiple interactions with renin residues may have contributed to the higher inhibitory activity when compared to activities of TF and LY that formed less number of interactions. However, the three peptides did not bind well to the unique sub-pocket S3*^sp^* (Gln13, Tyr14, Val30, Tyr155, Ala218, Ser219, Ala303) of renin, which has been shown to be necessary for high affinity inhibition of enzyme activity [28]. This is not surprising since peptides have been shown to bind poorly to S3*^sp^* and peptide modification to improve lipophilic character was suggested to be necessary for enhanced binding [28]. Additionally, ligand binding to the catalytic aspartate residues Asp 32 or Asp 215 is vital for renin inhibition [4]. The three peptides used in this study showed H-bond interactions with Asp 215 (Table 4), and the length of H-bond formed between Asp 215 and peptides were 2.32 Å, 2.46 Å and 2.19 Å for TF and LY RALP, respectively (Table S2). Moreover, RALP formed strong hydrogen bond network with the amino acids Gly34, Asn37, Arg74, Ser76, and Asp215 present within the active site of renin, while TF and LY formed relatively weak H-bond with residues Ser76, Thr77 and Asp215 (Table S2). Thus, overall the molecular docking data are consistent with the higher renin inhibition by RALP when compared to inhibitory activities of LY and TF. However, Aliskiren forms similar number of H-bond interactions (Table S2) but is closer to more renin active site residues (Table 4) and thus has higher renin-inhibitory potency than RALP.

## Conclusion

In the present work, the interactions of TF, LY and RALP with ACE or renin have been successfully characterized using fluorescence spectra, CD spectra and molecular docking techniques in combination with enzyme inhibition kinetics. The *in vitro* data clearly revealed that LY is a better inhibitor of ACE while RALP is a better renin inhibitor. The higher ACE-inhibitory activity of LY could be attributed to a higher collision rate constant and ability to convert the ACE protein into a more disordered structure. The mechanism responsible for the high renin-inhibitory activity of RALP probably involved the ability to form higher number of H-bonds with active site residues as well as the extensive reductions in α-helix and β-sheet fractions, which also resulted in a highly disordered enzyme protein structure. Results from this work also suggest that the presence of a positively-charged residue in RALP may have enhanced multiple interactions with negatively charged amino acid residues close to renin active site, which could have contributed to increased peptide inhibitory activity.

## Supporting Information

Table S1 **Predicted binding energies (Electrostatic energy: Eele; Van der Waals energy: Evdw; Potential energy: Epot, kJ/mol).**
(DOC)

Table S2 **Hydrogen bonds observed between ACE (PDB: 1O86) or renin (PDB: 2V0Z) and the docked top ranked pose of peptides.**
(DOC)

Table S3 **The distance between Zn^2+^ coordination amino acid residues within ACE (PDB: 1O86) and bioactive peptides.**
(DOC)
