# Supplementary material for: Evaluating Molecular Mechanism of Hypotensive Peptides Interactions with Renin and Angiotensin Converting Enzyme
Source: PLoS One. 2014 Mar 6;9(3):e91051. doi: 10.1371/journal.pone.0091051 (PMC3946342; doi:10.1371/journal.pone.0091051)
Supplement: Table S1 — Predicted binding energies (Electrostatic energy: Eele; Van der Waals energy: Evdw; Potential energy: Epot, kJ/mol). (DOC) [file pone.0091051.s001.doc]

**Table S1** Predicted binding energies (Electrostatic energy: *E_ele_*; Van der Waals energy: *E_vdw_*; Potential energy: *E_pot_* , kJ/mol)

| Ligand | ACE (PDB: 1O86) | | | Renin (PDB: 2V0Z) | | |
| --- | --- | --- | --- | --- | --- | --- |
|  | *E_ele_* | *E_vdw_* | *E_pot_* | *E_ele_* | *E_vdw_* | *E_pot_* |
| TF | -35.67 | -2.53 | -31.36 | -35.74 | -2.52 | -31.46 |
| LY | -43.03 | -4.56 | -39.21 | -43.06 | -4.81 | -39.91 |
| RALP | -214.25 | -12.42 | -192.81 | -212.28 | -13.12 | -192.88 |
| Lisinopril (ACE)/ Aliskiren (Renin) | -90.88 | -7.10 | -75.82 | -63.31 | -10.84 | -42.89 |
