# Supplementary material for: Evaluating Molecular Mechanism of Hypotensive Peptides Interactions with Renin and Angiotensin Converting Enzyme
Source: PLoS One. 2014 Mar 6;9(3):e91051. doi: 10.1371/journal.pone.0091051 (PMC3946342; doi:10.1371/journal.pone.0091051)
Supplement: Table S2 — Hydrogen bonds observed between ACE (PDB: 1O86) or renin (PDB: 2V0Z) and the docked top ranked pose of peptides. (DOC) [file pone.0091051.s002.doc]

**Table S2** Hydrogen bonds observed between ACE (PDB: 1O86) or renin (PDB: 2V0Z) and the docked top ranked pose of peptides

| ACE residues in H bonds | Number of H-bonds and their corresponding distance (Å) | | | | Renin residues in H bonds | Number of H-bonds and their corresponding distance (Å) | | | |
| --- | --- | --- | --- | --- | --- | --- | --- | --- | --- |
|  | TF | LY | RALP | Lisinopril |  | TF | LY | RALP | Aliskiren |
| Glu162:OE1 |  |  |  | 1:2.23 | Tyr14: |  |  |  | 1:2.25 |
| Gln281: HE21 |  | 1: 2.17 | 2:2.26, 2.12 |  | Asp32:OD1 |  |  |  | 1:2.04 |
| Gln281: HE22 |  |  |  | 2:2.41, 2.18 | Asp32:OD2 |  |  |  | 1:2.16 |
| Ala354:O | 1:2.16 | 1:2.34 |  | 1:2.04 | Gly34:O |  |  | 1:2.34 | 1:1.99 |
| Ala354: HN |  |  |  | 1:2.01 | Asn37:OD1 |  |  | 1:1.79 |  |
| Glu384:OE1 |  | 1:2.14 |  |  | Arg74:O |  |  | 1:2.17 | 1:1.99 |
| Glu384:OE2 | 1: 2.12 | 1:2.27 |  | 1:2.48 | Ser76:HN | 1:1.89 |  | 2:2.05, 2.11 | 1:2.11 |
| Asp415:OD2 |  |  | 1:2.25 |  | Ser76:HG | 2:2.10, 2.06 | 1:2.09 | 1:2.91 |  |
| Lys511:HZ1 |  |  |  | 1:1.93 | Thr77:HN | 1:2.43 |  |  |  |
| Tyr520: HH |  |  | 1:2.43 | 1:2.06 | Thr77:HG1 | 1:1.93 | 1:2.03 |  |  |
| Tyr523:OH |  |  |  | 1:2.42 | Gln128:O |  |  | 1:1.90 |  |
|  |  |  |  |  | Asp215:OD1 | 1:2.32 | 1:2.46 | 1:2.19 | 1:2.11 |
|  |  |  |  |  | HOH184:H2 |  |  |  | 1:2.02 |
| Total | 3 | 4 | 4 | 9 |  | 6 | 3 | 8 | 8 |
