# Supplementary material for: Evaluating Molecular Mechanism of Hypotensive Peptides Interactions with Renin and Angiotensin Converting Enzyme
Source: PLoS One. 2014 Mar 6;9(3):e91051. doi: 10.1371/journal.pone.0091051 (PMC3946342; doi:10.1371/journal.pone.0091051)
Supplement: Table S3 — The distance between Zn2+ coordination amino acid residues within ACE (PDB: 1O86) and bioactive peptides. (DOC) [file pone.0091051.s003.doc]

**Table S3** The distance between Zn^2+^ coordination amino acid residues within ACE (PDB: 1O86) and bioactive peptides

| Residues and ligand | Distance (Å) involved in coordination of Zn^2+^ | | | |
| --- | --- | --- | --- | --- |
|  | TF | LY | RALP | Lisinopril |
| His 383 NE2 | 2.037 | 2.037 | 2.037 | 2.037 |
| His 387 NE2 | 2.071 | 2.071 | 2.071 | 2.071 |
| Glu 411 OE1 | 1.995 | 1.995 | 1.995 | 1.995 |
| TF O14 | 2.433 |  |  |  |
| LY O15 |  | 2.210 |  |  |
| RALP O10 |  |  | 2.209 |  |
| Lisinopril O2 |  |  |  | 2: 2.321 |
